# Persistence of Primitive Reflexes as Possible Predictive Factors for Progression, Prevention, and Early Rehabilitation Intervention in Idiopathic Scoliosis

**DOI:** 10.3390/medicina61030427

**Published:** 2025-02-28

**Authors:** Liliana Vlădăreanu, Mădălina Gabriela Iliescu, Iulia Tania Andronache, Elena Danteș

**Affiliations:** 1 Doctoral School of Medicine, “Ovidius” University of Constanta, 900470 Constanta, Romania; liliana.vladareanu@365.univ-ovidius.ro (L.V.); elena.dantes@365.univ-ovidius.ro (E.D.); 2 Rehabilitation Department, Faculty of Medicine, “Ovidius” University of Constanta, 1 University Alley, 900470 Constanta, Romania; 3 Pediatric Neurorehabilitation Department, Techirghiol Balneal and Rehabilitation Sanatorium, 34-41 Climescu Blvd., 906100 Techirghiol, Romania; 4 Department of Rheumatology, Internal Medicine Clinic, “Alexandru Gafencu” Military Emergency Hospital Constanta, Mamaia Blvd., 900527 Constanța, Romania; andronacheiulia@gmail.com; 5 Clinical Hospital of Plmonology, 40 Sentinelei Street, 900002 Constanta, Romania

**Keywords:** idiopathic scoliosis, retained primitive reflexes, early scoliosis detection, early scoliosis rehabilitation

## Abstract

*Background and objectives*: Idiopathic scoliosis is a three-dimensional spinal deformity characterized by a lateral curvature exceeding 10 degrees in the frontal plane accompanied by vertebral rotation in the transverse plane. Despite extensive research on genetic and neurological factors, its etiology is uncertain. This prospective observational study aims to investigate the relation between the primitive reflexes, specifically, the asymmetric tonic neck reflex (ATNR), symmetric tonic neck reflex (STNR), and spinal Galant reflex (SGR), which play key roles in early motor development and postural control and the severity of idiopathic scoliosis (measured via the Cobb angle and the Nash–Moe rotational quota. Additionally, the study evaluated whether the retention of primitive reflexes correlates with increased progression risk over 12 months of conservative treatment. *Materials and Methods*: Our study cohort included 162 patients, aged 7–19 years, diagnosed with idiopathic scoliosis, who underwent clinical examination and assessment of retained primitive reflexes using standardized grading systems. *Results*: A total of 162 patients (95 girls, 67 boys; mean age: 12.73 ± 2.74 years) met the inclusion criteria. In 73.5% of the cases, scoliosis was detected, with the majority occurring in the dorsal region (40.1%). The mean initial Cobb angle was 13.49° ± 7.14°, with no significant change after 12 months of conservative treatment (*p* = 0.584). Nash–Moe rotation scores were 1 in 52.5% and 2 in 22% of the cases. Retention of the following primitive reflexes were identified at baseline: Moro (19.1%), ATNR (38.3%), STNR (44.4%), and GSR (27.8%). GSR retention significantly correlated with the Cobb angle (*p* = 0.011; R = 0.233). All the reflex scores decreased significantly after 12 months, but no correlation existed between the retained reflexes and scoliosis progression. Patients with a history of quadrupedal locomotion had significantly lower ATNR (*p* = 0.002), STNR (*p* < 0.001), and GSR (*p* = 0.017) retention. *Conclusions*: These findings suggest that primitive reflex testing could serve as an early screening tool in scoliosis risk stratification, being a cost-effective, non-invasive instrument for identifying at-risk children before clinically significant deformity develops.

## 1. Introduction

Idiopathic scoliosis represents the apparition of a non-physiological curvature in the frontal plane of the spine that exceeds 10° Cobb angle, as measured on a standard posterior–anterior stitching radiograph, and associated with rotation in the spinal transversal plane. As an exclusion diagnosis, it is obtained after all other spinal deformities, neurological diseases, and syndromic pathologies have been ruled out [1]. Those of adolescent age, ranging from 10 to 19 years old, are the most affected population, as adolescent idiopathic scoliosis (AIS) is the most prevalent of idiopathic scoliosis, with a global incidence within the range of 2–5.2% [2]. Treatment for idiopathic scoliosis can be conservative, for cases with a Cobb angle under 45°, or surgical, for cases with a Cobb angle exceeding 45 ° [3]. Conservative treatment consists of physiotherapeutic scoliosis-specific exercises (PSSEs) when the Cobb angle is under 30° and PSSE and rigid bracing when the Cobb angle exceeds 30° [3,4]. Conservative treatment for adolescent and all-age patients, as well as their families, is time-consuming and resource-intensive, requiring specialized centers typically located in big cities. Teenage patients also tend to consider rigid bracing hard to accept as a treatment for psychological reasons, and as a result, they have low compliance with the conservative treatment, which is a reason for Cobb angle progression, leading to surgical treatment. The mean age for an idiopathic scoliosis-positive diagnosis is 9–10 years old, with no major gender differences.

Research in the last few decades has increasingly pointed toward a genetic and neurodevelopmental etiopathogenesis for AIS, but genetic testing is still expensive and mainly available only in research facilities, which are, again, concentrated in big cities [5,6,7].

The easiest way to determine neurodevelopmental age is to check the integration of primitive reflexes, as neonate specialists already use this method, which is standardized. Primitive reflexes are automatic motor responses encoded in our deoxyribonucleic acid (DNA) that enable us, as a species, to develop motor skills [8,9,10]. Out of the primitive reflexes that are regularly tested, the ones that involve rotation and lateral flexion of the trunk and spine are the asymmetric tonic neck reflex (ATNR), the symmetric tonic neck reflex (STNR), and the spinal Galant reflex (SGR). These reflexes are needed for the newborn and toddler to start crossing the vertical and horizontal midlines to achieve efficient trunk rotation, which is necessary for side rolling and, later, crawling [11,12]. Of the three previously described primitive reflexes, the first to appear and integrate is the ATNR, followed by the GSR and STNR, in that order, with none remaining physiologically after than 12 months of age. For some children, due to unknown causes and in the absence of any neurological pathology, these reflexes tend to still be active, even if their age is over 12 months.

This prospective observational study intended to check if, in a population of children with idiopathic scoliosis, there was any significant incidence of retained primitive reflexes in the form of a retained Moro reflex, ATNR, STNR, or GSR, and if there was any correlation between the Cobb angle value, the Nash–Moe rotational quota, and the retention rate of the abovementioned primitive reflexes. Also, the study tried to evaluate if there was a statistically relevant difference in Cobb angle progression for the population with retained primitive reflexes.

If any or more of the hypotheses described above are found to be true, it will open up a window of opportunity for future research to try and develop early diagnostic tools that are cheap and reliable and that can be used, with minimal personnel training, to effectively determine, before adolescence, the risk of developing scoliosis and Cobb angle progression.

## 2. Materials and Methods

### 2.1. Ethical Approval

Informed consent was obtained in writing from all participants for the procedures and the use of their medical data. For individuals aged 16 and above, consent was taken solely from the patients. For those under 16, consent was obtained from both the patients and their parents. Ethical approval for this study was granted by the medical unit where the research was developed, with ethical form number 3739/11.03.2022 and 19377/08.12.2022.

### 2.2. Subjects

Over a 30-month period, from June 2022 to December 2024, we evaluated a total of 443 patients from Pediatric Rehabilitation Department of Techirghiol Hospital, Romania, with spinal pathology. From this group, using the inclusion/exclusion criteria mentioned below, we selected data from 162 patients, which included 95 girls and 67 boys ranging in age from 7 to 19 years. These patients attended the clinic for the evaluation and treatment of developmental vertebral disorders, specifically idiopathic scoliosis.

### 2.3. Methodology

A prospective observational study was conducted to analyze patient data collected over a 30-month period. Out of 443 patients, including both adults and children, only 160 patients were selected based on the inclusion criteria outlined below.

#### 2.3.1. Inclusion Criteria

- Age under 19 years.
- Primary vertebral disorders—idiopathic scoliosis—as the main diagnostic.
- Written consent for the use of personal data for scientific analysis.

#### 2.3.2. Exclusion Criteria

- Age above 19 years.
- Personal positive medical history for neurological, congenital genetic, or orthopedic pathology.
- Lack of written consent for scientific personal data analysis.

#### 2.3.3. Features of the Clinical Standard Evaluation of These Patients

- Adam’s test—standing and seated [13].
- Finger-to-toe index (FTI)—standing and supine, measured in centimeters (cm) [14].
- Moro, ATNR, STNR, and GSR testing for children older than 12 months, using the Ayres grading system from 0 to 4 [11,15,16] with two independent evaluators, each with a minimum of three years of experience; when the evaluation results for the same patient were different, a third evaluator was asked to perform a clinical examination, and these results were the ones taken into consideration for the study. One initial evaluation was performed at the start of the treatment and another final evaluation was performed after 12 months of specific treatment.

#### 2.3.4. The Anamnestic Standard Evaluation

During the evaluation, the patients/their parents had to complete a medical history questionnaire that included information about the following:

- Motor acquisition in the first year of life, specifically crawling between the ages of 6 to 9 months, which were recorded as positive, negative, or “I do not know/I do not remember”.
- Dioptric correction (emmetrope, myopic, hypermetropic, astigmatism).
- Positive/negative history of associated pathologies, as mentioned in the exclusion criteria.

#### 2.3.5. Imagistic Evaluation

The study included standard standing spine radiographs taken from posterior–anterior and lateral views, following the SOSORT guidelines [3] and international consensus [17,18]. The following measurements and evaluations were conducted:

- Cobb angle: measured in degrees (°), with values above 10° indicating scoliosis; one initial measurement and one final one after 12 months of treatment.
- Nash–Moe rotational quota: rated on a scale from 0 to 4; one initial measurement and one final one after 12 months of treatment [17] (https://www.srs.org/Files/Research/Manuals-and-Publications/sdsg-radiographic-measuremnt-manual.pdf) (accessed on February 2022)
- The Risser Index, which assesses iliac crest ossification maturity, scored from 0 to 5 [17] (https://www.srs.org/Files/Research/Manuals-and-Publications/sdsg-radiographic-measuremnt-manual.pdf)(accessed on February 2022)
- Iliac crest asymmetry: measured in millimeters (mm).
- Congenital spinal malformations that would exclude the patient from the study.

### 2.4. Data Analysis Methodology

All the data from the study were analyzed using IBM SPSS Statistics 25 and illustrated using Microsoft Office Excel/Word 2024. Quantitative variables were tested for normal distribution using the Shapiro–Wilk test and were written as averages with standard deviations or medians with interquartile ranges. Quantitative independent variables with non-parametric distributions were tested between groups using the Mann–Whitney U test. Correlations between quantitative independent variables with non-parametric distributions were measured using Spearman’s rho correlation coefficient. Quantitative variables with non-parametric distributions measured at the initial and final check-ups were tested between measurements using the Wilcoxon test. Univariable and multivariable logistic regression models were used to predict scoliosis grading improvement. The models were tested for goodness-of-fit and significance. The prediction’s performance was estimated using odds ratios with 95% confidence intervals and significance values.

Qualitative variables were written as counts or percentages. The threshold considered for the significance level for all tests was α = 0.05.

## 3. Results

### 3.1. Lot Statistical Analysis

The refined lot used for this paper had the following characteristics, as shown in Table 1.

The results show that the mean age was 12.73 ± 2.74 years (median = 13 years, IQR = 10.75–14), and most patients that were evaluated were girls (58.6%). Out of the 160 patients, 73.5% had scoliosis according to the Cobb classification; most of these were dorsal (40.1%), of which 60 (37%) were right dorsal and 5 (3.1%) were left dorsal. Dorso-lumbar scoliosis was found in 29% of the other patients, with 42 patients exhibiting this on the right side and 1 patient on the left. The Nash–Moe rotational value in the lot was 1 for 52.5%, and the Risser Index osseous maturation was 3 for 46.6%. The Adam’s test was positive for 119 patients, with 67.3% on the right side and 6.2% on the left side. Thirty-four individuals in the scoliotic population experienced ocular conditions, with 17.3% having astigmatism and 3.7% having myopia. The mean finger–floor distance was 18.8 ± 9.38 cm (median = 15, IQR = 15–25). The stabilometry plantigrade testing resulted in an abnormal measurement for 46 patients (28.4%), and stabilometry digitigrade testing resulted in an abnormal measurement for 128 patients (79%). A positive history of quadrupedal locomotion in infancy was present in 50.7% (76) of the 150 patients.

### 3.2. Evolution of Retained Reflexes During Evaluation and Treatment

At the initial evaluation, out of the 162 patients in the lot, 131 had integrated the Moro reflex (Ayres 0), 18 were scored as Ayres 1, 9 as Ayres 2, 1 as Ayres 3, and 1 as Ayres 4.

**Figure 1:**
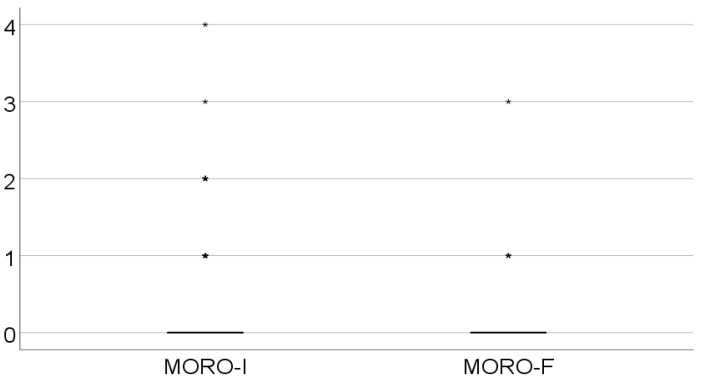
Evolution of the retained Moro reflex.

The Moro score was significantly lower in evolution (*p* < 0.001), and the mean difference between measurements was 0.23 ± 0.55, while the median = 0 (IQR = 0–0), as shown in Table 2 and Figure 1.

**Figure 2:**
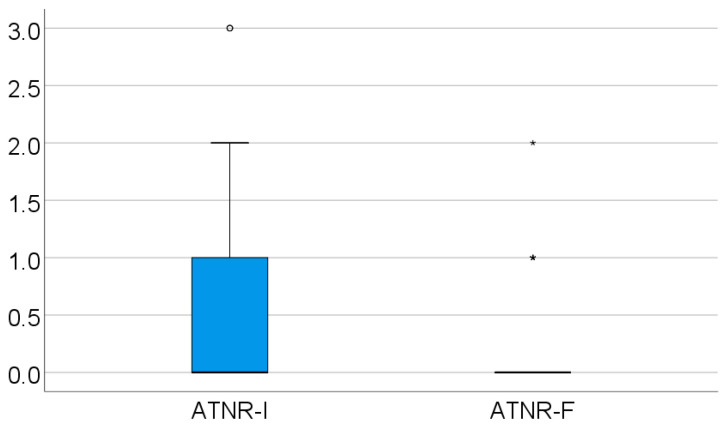
Evolution of the retained ATNR.

In the studied group for the initial evolution of the ATNR, out of all the patients, 100 had a score of 0 Ayres, 46 had a score of 2 Ayres, 14 had a score of 3 Ayres, and none had a score of 4 Ayres. The ATNR score was significantly lower in evolution (*p* < 0.001), and the mean difference between measurements was 0.46 ± 0.64, with the median = 0 (IQR = 0–1), as shown in Table 3 and Figure 2.

For the STNR, at the initial evaluation, 92 patients tested as 0 on the Ayres scale, while 53 tested as 1, 16 tested as 2, 1 tested as 3, and none tested as 4 on the Ayres scale.

**Figure 3:**
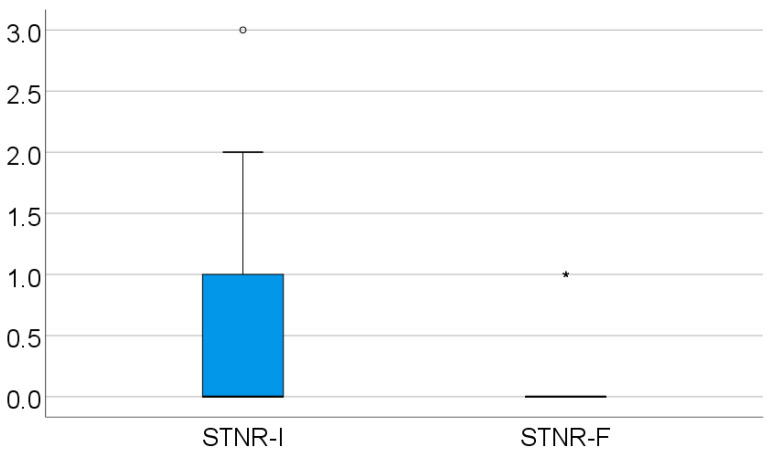
Retained STNR evolution.

For this reflex, most positives were found with values 1 and 2 on the Ayres scale, compared with the other tested primitive reflexes. The second in the total number of positive findings is the ATNR. The STNR score was significantly lower in evolution (*p* < 0.001), with a mean difference between measurements of 0.46 ± 0.64 and a median = 0 (IQR = 0–1), as shown in Table 4 and Figure 3.

**Figure 4:**
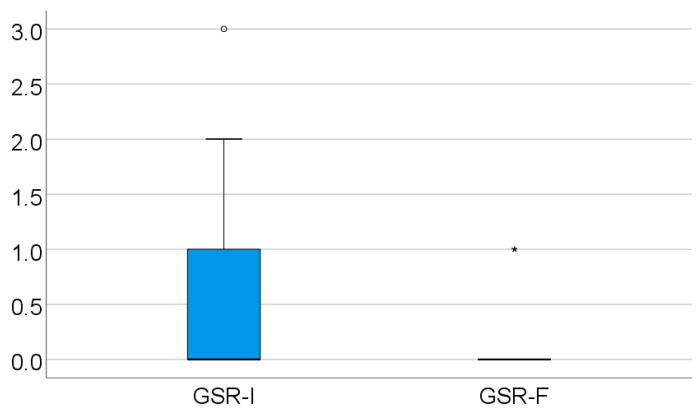
Retained GSR evolution.

For the GSR, the initial evaluation showed 117 patients with a zero value on the Ayres scale, 28 patients with a value of 1, 16 patients with a value of 2, 1 patient with a result of 3, and none with a result of 4 on the Ayres scale. The GSR was the third most retained reflex from the ones tested in the study, after the STNR and ATNR. The GSR score was significantly lower in evolution (*p* < 0.001), with a mean difference between measurements of 0.37 ± 0.65 and a median = 0 (IQR = 0–1), as can be seen in Table 5 and Figure 4.

### 3.3. Cobb Angle Evolution

According to Table 6, the Cobb angle evolution did not differ significantly in evolution (*p* = 0.584). In this plot, most of the patients (84) had mild scoliosis, and only 7 had severe scoliosis, according to the SOSORT classification.

Data from Table 7 shows the distribution of the patients according to their scoliosis grading based on the Cobb angle (before and after treatment). The results show that most of the patients (91 patients; 77.11%) had maintained their scoliosis grading, while 8 (6.77%) patients had lowered their scoliosis grading (the treatment having an improving effect), and for 17 patients (14.4%), their scoliosis grading had increased; however for 10 patients, the difference between pre- and post-treatment was only 2 degrees for the Cobb angle (from 8 or 9 degrees to 10 degrees, reclassifying them from grade 0 to grade 1; an insignificant change).

Data from Table 8 shows the comparison of Moro, ATNR, STNR and GSR evolution differences in patients according to the existence of scoliosis grading improvement. According to the results, only the Moro score difference (*p* = 0.020) and the STNR difference (*p* = 0.032) were significantly different between patients that showed or did not show an improvement in scoliosis grading. In this study, patients that had an improvement had a significantly higher value for the Moro score evolution difference (median = 0.5, IQR = 0–2) and STNR evolution difference (median = 1, IQR = 1–1) in comparison with patients without improvement (Moro: median = 0, IQR = 0–0; STNR: median = 0, IQR = 0–1).

Data from Table 9 shows the univariable and multivariable logistic regression models used to predict scoliosis grading improvement. In the univariable models, the Moro difference (*p* = 0.010) and the initial Cobb angle (*p* = 0.029) were significant predictors, while the GSR difference was not significant in prediction (*p* = 0.422). Using a multivariable regression model that included the Moro difference while using age, gender, and initial Cobb angle as adjusting covariates, the data show that the Moro difference remains a significant predictor; as such, patients that had an improvement of 1 point in the Moro score at 12 months after treatment tan increased their odds of having a scoliosis grading improvement by 2.809 times (95% C.I.: 1.018–7.754; *p* = 0.046).

**Figure 5:**
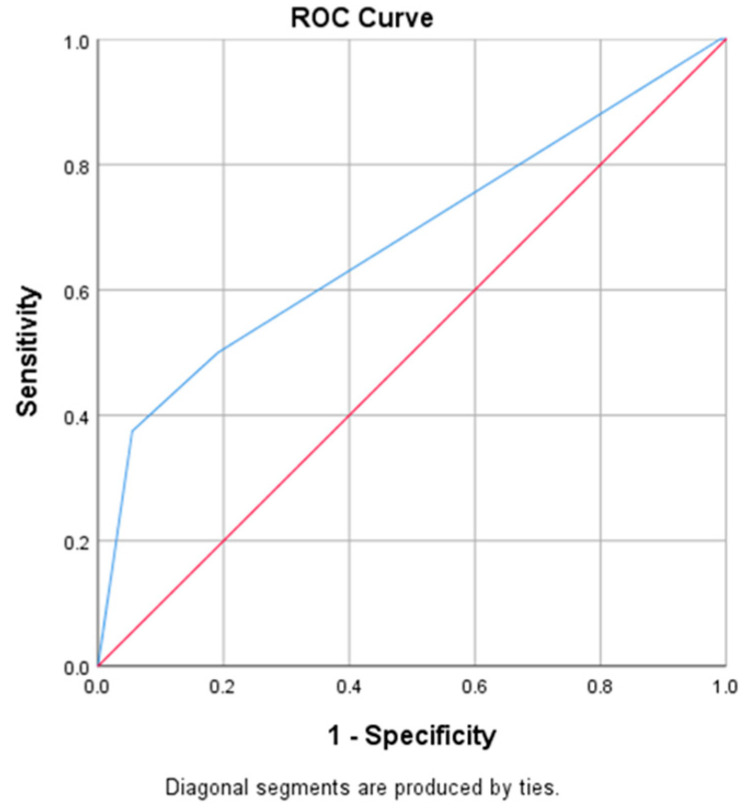
ROC curve in establishing the cut-off value for the Moro score evolution difference in scoliosis grading improvement.

Data from Table 10 and Figure 5 show the ROC curve in establishing the cut-off value of the Moro score evolution difference in scoliosis grading improvement. As the results show, the usage of an ROC curve in this study is not adequate for this parameter because of the small number of patients that had an improvement in scoliosis grading (N = 8). Because of the small number of possibilities and the difference in in the values observed for the Moro score (ranging from −1 to 2), the results show that there is a tendency toward statistical significance in the direction of a significant prediction using the ROC curve.

### 3.4. Correlations Between the Initial Value of the Cobb Angle and Moro, ATNR, STNR, and GSR Initial Values

**Figure 6:**
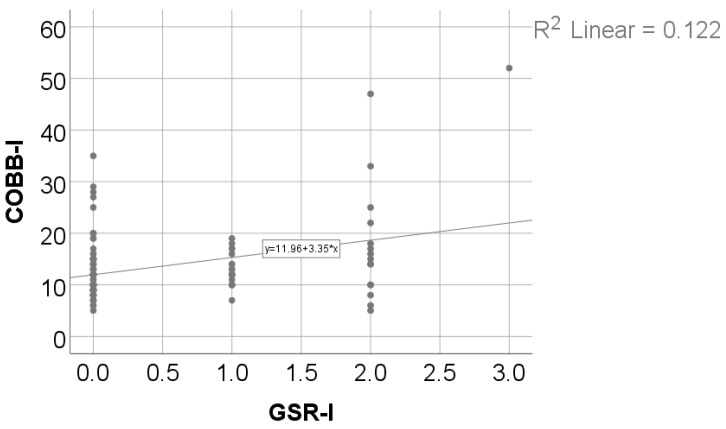
Correlation between initial values of the Cobb angle and the retained GSR.

Data from Table 11 and Figure 6 show the correlations between the initial value of the Cobb angle and Moro, ATNR, STNR, and GSR initial values. Most of the correlations investigated were not statistically significant (*p* > 0.05), except for the correlation between the Cobb angle and the GSR (*p* = 0.011, R = 0.233). This significant positive low-power correlation shows that patients with higher initial values for the Cobb angle were significantly more associated with higher initial values for the GSR and vice-versa.

### 3.5. Correlation Between the Initial Value of the Cobb Angle and Moro, ATNR, STNR, and GSR Evolution Differences

**Figure 7:**
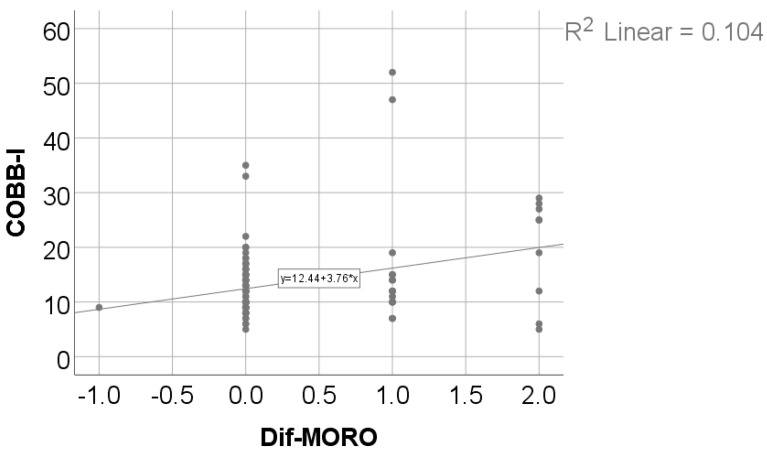
Correlation between initial values of the Cobb angle and the Moro score evolution difference.

**Figure 8:**
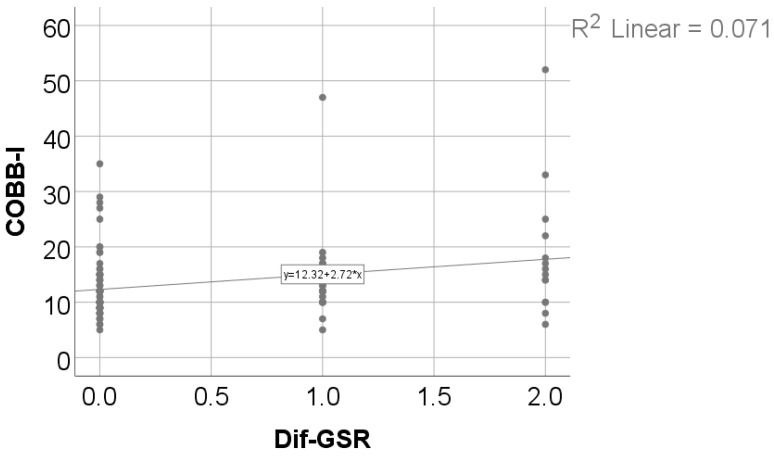
Correlation between initial values of the Cobb angle and the GSR score evolution difference.

Data from Table 12 and Figure 7 and Figure 8 show the correlations between the initial value of the Cobb angle and the Moro, ATNR, STNR, and GSR evolution differences. Most of the investigated correlations were not statistically significant (*p* > 0.05), except for the correlations between the initial value of the Cobb angle and the Moro score evolution difference (*p* = 0.028, R = 0.203) or GSR evolution difference (*p* = 0.011, R = 0.234); these significant positive low-power correlations show that patients with higher initial values for the Cobb angle were significantly more associated with higher decreases from the initial values for the Moro or GSR score and vice-versa.

### 3.6. Comparison of Initial Values of Moro, ATNR, STNR and GSR Scores According to the Existence of Quadrupedal Locomotion in Infancy

**Figure 9:**
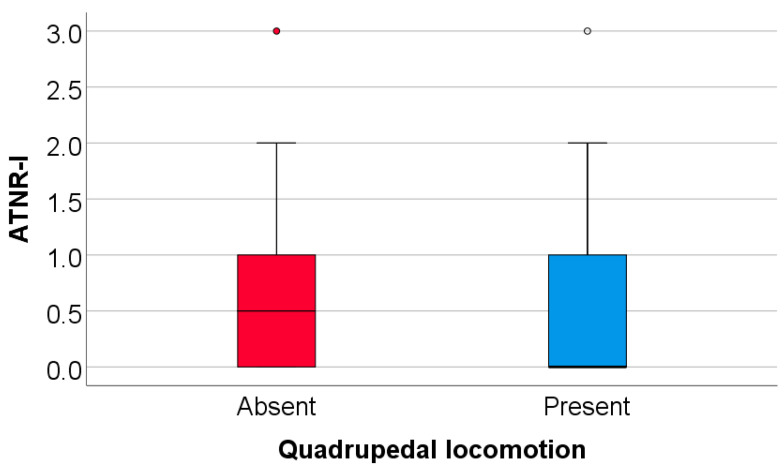
Comparison of initial values of the ATNR according to the existence of quadrupedal locomotion.

**Figure 10:**
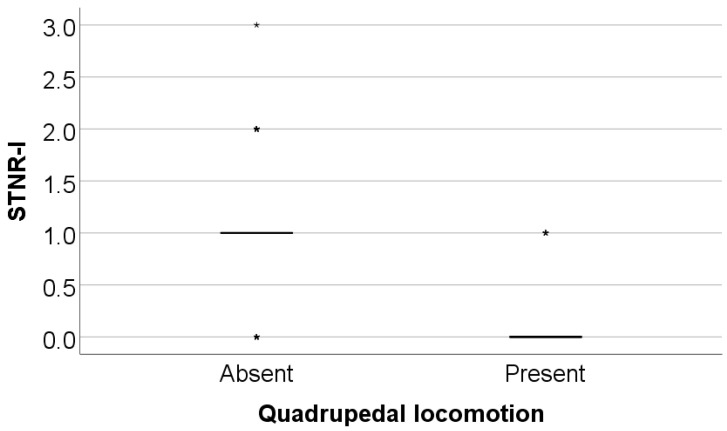
Comparison of initial values of the STNR according to the existence of quadrupedal locomotion.

**Figure 11:**
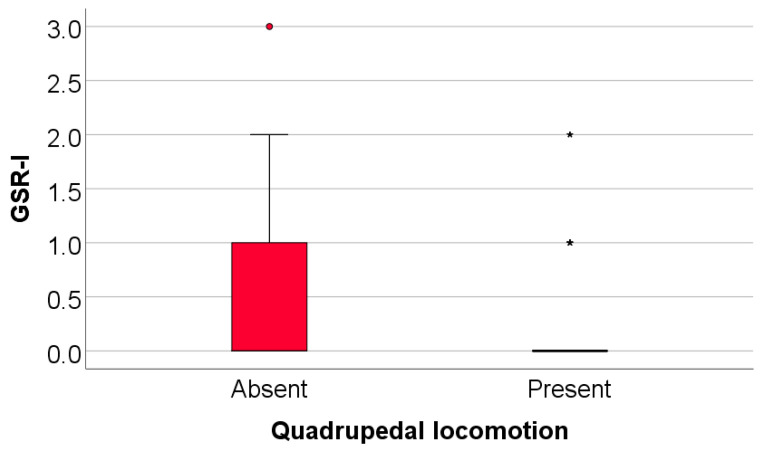
Comparison of initial values of the GSR according to the existence of quadrupedal locomotion.

Data from Table 13 and Figure 9, Figure 10 and Figure 11 show the comparison of initial values of Moro, ATNR, STNR, and GSR scores according to the existence of quadrupedal locomotion in infancy.

The Moro score was not significantly different between groups (*p* = 0.851); however, the ATNR score (*p* = 0.002), STNR score (*p* < 0.001), and GSR score (*p* = 0.017) were substantially lower in patients who had quadrupedal locomotion in comparison with patients without quadrupedal locomotion (ATNR: median = 0, IQR = 0–1 vs. median = 0.5, IQR = 0–1; STNR: median = 0, IQR = 0–0 vs. median = 1, IQR = 1–1; GSR: median = 0, IQR = 0–0 vs. median = 0, IQR = 0–1).

## 4. Discussion

### 4.1. Study Motivation and Comparison with Published Data

Conservative treatment for idiopathic scoliosis consists of specific exercises and rigid bracing tailored for each patient. As per the Scoliosis Research Society (SRS) and International Society on Scoliosis Orthopedic and Rehabilitation Treatment (SOSORT) 2018 guidelines, an experienced scoliosis therapist should develop a conservative rehabilitation program, and the patient evaluation and bracing should be conducted by a skilled medical doctor using one of the seven recognized evidence-based methods [3,17,18,19]. Finding an experienced therapist and clinician is a complicated issue since the training needed to become a scoliosis therapist is still not widely available, so most specialists are concentrated in big cities or institutions, making access for the patient difficult. Furthermore, physical therapists’ training and knowledge about idiopathic scoliosis vary widely and depend on their experience, place of work, and other factors [20,21,22,23].

Also, even when braces and PSSE programs are developed using SRS or SOSORT recommendations, there is the problem of patients’ low compliance with conservative treatment, as most of the adolescent population find rigid bracing, which is most commonly prescribed, hard to accept and the physical exercise program time-consuming [24,25,26,27].

The aim of the conservative treatment for idiopathic scoliosis is to stop progression or to lower the Cobb angle value and the Nash–Moe rotational quota and keep them under 20° for the Cobb angle and under 2 for the Nash–Moe quota. Patients who reach bone maturity with a Cobb value of over 30° are at a higher risk of progression in adult life and at a higher risk of musculoskeletal pain and dysfunction.

All of the above are reasons are why, in order to prevent progression in the Cobb angle and the rotational quota in these patients, it is important to find an easy-to-use tool, both for the clinician and for the physical therapist, that is non-invasive and not harmful for the patient, easy and cheap to teach with and use for medical professionals, and that can help predict, as early as possible, the risk of developing a scoliotic curvature.

Primitive reflexes are currently used to determine the motor development level of newborns and infants and the motor and neurological development of toddlers everywhere in the world. They are widely used until the age of 3 years, which is 6 to 7 years earlier than the scoliosis screening. This early use underscores their crucial role in early detection. They are taught to all primary care clinicians during basic training, making them one of the most straightforward tools for potential use in scoliosis development.

There are no published studies that make a clear connection between scoliotic curvature and retained primitive reflexes, but there is sufficient literature that connects dyspraxias—developmental coordination disorders—with retained primitive reflexes and inappropriate postural control [8,9,10,11,12,28,29,30,31,32].

### 4.2. Predicted Versus Real Findings in the Evolution of Retained Primitive Reflexes

Primitive reflexes are complex, automatic response movement patterns that are mediated by the brainstem. As the central nervous system matures, these reflexes become harder to elicit after six months, when voluntary motor activity starts and cortical inhibition begins [11]. The Moro reflex, ATNR, STNR, and GSR (truncal incurvation reflex) are the reflexes we tested and included in our evaluation. In an infant/child with normal neurodevelopment, these reflexes are later replaced by postural reactions that are involved in the motor acquisition of hand–eye coordination, sitting, crawling, 3-D orientation, and locomotion. As mentioned before, there are numerous published studies that connect idiopathic scoliosis with a modified postural control response [30,31,32].

Our predicted results were expected to show that there would be no significant difference in evolution for the Moro (related to limb flexion–extension due to rapid cervical spine flexion–extension) and the STNR (related to independent movements between the upper and lower limbs and related directly to crawling) and a significant difference in evolution for the ATNR (related to independent movements between the right upper and lower limbs and the left upper and lower limbs due to cervical spine rotation) and the GSR (related to thoracolumbar side-bending due to cutaneous paravertebral stimulation). We based our prediction on the idea that treating the scoliotic curvature would provide patients with better frontal plane coordination at the spine and limb level.

Our study showed that all four reflexes had a significantly lower value in evolution, confirming, yet again, the idea that considering only the frontal plane when treating scoliotic curves is not enough. Also, as the evolution of the Cobb angle was not significantly different in the retained primitive reflexes groups, we cannot possibly say that there was a difference in the patient’s progress in the group with the most retained primitive reflexes (STNR or ATNR).

### 4.3. Correlation Between the Initial Cobb Angle Value and Retained Primitive Reflexes

Our study showed that there was a significant correlation between the initial Cobb angle value and the value of the Ayres score for the GSR, meaning that the higher the Cobb, the higher the Ayres score was for the GSR and vice-versa. To test the GSR, the infant is in the prone position, and the evaluator must touch the paravertebral region starting from the inferior angle of the scapula (7th–8th thoracic vertebra) toward the lumbar area (2nd–3rd lumbar vertebra), using, as a trigger, finger pressure or a neurological hammer. The motor response consists of a concavity of the trunk on the same side as the trigger [11,33,34]. This result aligns with the prediction for our study, as the GSR is related to frontal plane spinal movements. Testing for an older child or an adolescent does not vary compared to the evaluation of the GSR in infants, and as described above, it is easy to perform in the general practitioner’s office and school medical office if necessary, making it an easy tool to use in screening.

Another finding related to the initial Cobb angle was that there was a significant positive low-power correlation between the patients with higher initial values and higher decreases from the Ayres initial scores for the Moro reflex and the GSR. For the Moro reflex, this may be related to the active extension of the upper limb and spine postures used in the kinetic conservative treatment of scoliosis, which warrants further investigation.

### 4.4. Quadrupedal Locomotion in Infancy and Retained Primitive Reflexes

In the literature, quadrupedal locomotion in infancy is related to the ATNR (asymmetrical limb extension–flexion of the limbs involving the frontal plane) and the STNR (symmetrical limb extension–flexion of the limbs in the transverse plane), and usually, children who do not go through the crawling phase as infants tend to have retained the ATNR and the STNR. Since using an anamnestic questionnaire is easy and requires almost no extra resources in a medical office, we thought that adding this question about crawling history might give us an insight into the risk of still having retained the ATNR and STNR as a child or adolescent. And since crawling occurs between 9 and 12 months in the normal neurodevelopment of infants, if it is related to these two reflexes, it might give us an inexpensive tool to determine which children to screen sooner for scoliotic curvatures.

Our results showed that not only were ATNR and STNR Ayres scores substantially lower in patients with quadrupedal locomotion in infancy, the GSR Ayres score was also substantially lower for these patients.

### 4.5. Study Limitations and Potential Research

One of the main limitations of our study is the age of presentation of the patients in the lot as the youngest was 7 years old. It would be more precise to have a batch that can be evaluated from as young as 2–3 years old and later followed up to see if any who retain primitive reflexes do or do not develop a scoliotic curvature. This would allow us to establish a prospective research study that spans a more extended period.

Another limitation is that we only had available for the study four independent assessors who were experienced enough in testing retained reflexes in older children. This fact limited our batch considerably since it was a single-centered study.

Also, the number was even lower for therapists and evaluators trained at schools for the conservative treatment of idiopathic scoliosis and the evaluation of retained primitive reflexes. We only had two assessors that matched these criteria, further limiting our experiment.

Finding or training more specialists in these two subjects might lead to multi-centered research that can span more prolonged periods, yielding statistically more relevant results.

## 5. Conclusions

For the studied batch, there was no difference in the evolution of the Cobb angle value during conservative treatment as per SOSORT and SRS guidelines in relation to their initial Ayres score. During conservative scoliosis treatment, the Ayres scores significantly decreased for all the tested primitive reflexes: Moro, ATNR, STNR, and GSR. The higher the initial Cobb angle, the higher the initial Ayres score for the GSR. Patients who had quadrupedal locomotion as infants had a significantly lower Ayres score for the ATNR, STNR, and GSR.

Routine testing of retained reflexes for the ATNR, STNR, and GSR in younger children (ages 3 to 8 years old) might be a predictor for the development of a lateral curvature of the spine. It is cost-effective, and the exercises needed for their integration are easy to introduce not only into rehabilitation programs but also into school physical programs for all ages as prevention. Routinely examining the history of quadrupedal locomotion during infancy, as well as the family history of scoliosis, can assist the clinician in determining which segment of the pediatric population may require more frequent or earlier physical examinations.

More studies need to be conducted into finding a non-invasive, cheap, and easy-to-use predictor for developing scoliosis in the general population, as prevention remains the most cost-effective tool for idiopathic scoliosis, and the least intrusive in the day-to-day life of the patient and their families.

## 6. Patents

There are no patents pending or used for this prospective observational research.

## Figures and Tables

**Table 1:** Characteristics of the analyzed batch.

Parameter	Value
Age (Mean ± SD, Median (IQR))	12.73 ± 2.74, 13 (10.75–14)
Gender (Male) (Nr., %)	67 (41.4%)
Scoliosis—Location (Nr., %)	
Absent	43 (26.5%)
Dorsal	65 (40.1%)
Lumbar	7 (4.3%)
Dorso-lumbar	47 (29%)
Nash–Moe—Score (N = 118)	
0 points	23 (19.5%)
1 point	62 (52.5%)
2 points	26 (22%)
3 points	7 (5.9%)
Risser Index—Score (N = 118)	
0 points	3 (2.5%)
1 point	5 (4.2%)
2 points	32 (27.1%)
3 points	55 (46.6%)
4 points	22 (18.6%)
5 points	1 (0.8%)
Iliac crest symmetry (Mean ± SD, Median (IQR))	2.03 ± 4.75, 0 (0–0)
Cervical spine modifications (Nr., %)	83 (51.2%)
Adam test—Abnormal (Nr., %)	119 (73.5%)
Modified Adam test—Abnormal (Nr., %)	119 (73.5%)
Fukuda test—Positive (Nr., %)	23 (14.2%)
Motion sickness (Nr., %)	35 (21.6%)
Ocular status—Abnormal (Nr., %)	34 (21%)
Vestibular syndrome (Nr., %)	23 (14.2%)
Finger–floor distance (Mean ± SD, Median (IQR))	18.8 ± 9.38, 15 (15–25)
Stabilometry plantigrade test—Abnormal (Nr., %)	46 (28.4%)
Stabilometry digitigrade test—Abnormal (Nr., %)	128 (79%)
Quadrupedal locomotion (Nr., %) (N = 150)	76 (50.7%)

**Table 2:** Evolution of the retained Moro reflex.

Moro Score	Mean ± SD	Median (IQR)	*p* *
Initial value	0.28 ± 0.66	0 (0–0)	<0.001
Final value	0.05 ± 0.29	0 (0–0)	

* Related-samples Wilcoxon signed rank test.

**Table 3:** Evolution of the retained ATNR.

ATNR Score	Mean ± SD	Median (IQR)	*p* *
Initial value	0.49 ± 0.7	0 (0–1)	<0.001
Final value	0.04 ± 0.22	0 (0–0)	

* Related-samples Wilcoxon signed rank test.

**Table 4:** Retained STNR evolution.

STNR Score	Mean ± SD	Median (IQR)	*p* *
Initial value	0.54 ± 0.69	0 (0–1)	<0.001
Final value	0.02 ± 0.15	0 (0–0)	

* Related-samples Wilcoxon signed rank test.

**Table 5:** Evolution of the retained GSR.

GSR Score	Mean ± SD	Median (IQR)	*p* *
Initial value	0.39 ± 0.69	0 (0–1)	<0.001
Final value	0.02 ± 0.13	0 (0–0)	

* Related-samples Wilcoxon signed rank test.

**Table 6:** Cobb angle evolution.

Cobb Angle	Mean ± SD	Median (IQR)	*p* *
Initial value	13.49 ± 7.14	12 (10–15)	0.584
Final value	13.35 ± 6.87	12 (10–15)	

* Related-samples Wilcoxon signed rank test.

**Table 7:** Distribution of the patients according to their scoliosis grading based on the Cobb angle (before and after treatment).

Scoliosis Grade		After Treatment						
	N	Grade 0	Grade 1	Grade 2	Grade 3	Grade 4	Grade 5	Total
Before treatment	Grade 0	10	17	0	0	0	0	27
	Grade 1	4	78	0	0	0	0	82
	Grade 2	0	3	3	0	0	0	6
	Grade 3	0	0	1	0	0	0	1
	Grade 4	0	0	0	0	1	0	1
	Grade 5	0	0	0	0	0	1	1
	Total	14	98	4	0	1	1	118

Grade 0 = Cobb angle < 10 degrees; Grade 1 = Cobb angle of 10–24 degrees; Grade 2 = Cobb angle of 25–34 degrees; Grade 3 = Cobb angle of 35–44 degrees; Grade 4 = Cobb angle of 45–54 degrees; Grade 5 = Cobb angle of 55–64 degrees.

**Table 8:** Comparison of Moro, ATNR, STNR, and GSR evolution differences in patients according to the existence of scoliosis grading improvement.

Parameter/Improvement		Moro	ATNR	STNR	GSR
Absent	Meridian (IQR)	0 (0–0)	0 (0–1)	0 (0–1)	0 (0–1)
Present	Meridian (IQR)	0.5 (0–2)	0 (0–1)	1 (1–1)	0 (0–1.75)
*p* *		0.020	0.961	0.032	0.555

* Mann-Whitney U test.

**Table 9:** Univariable and multivariable logistic regression models used in the prediction of scoliosis grading improvement.

Parameter	Univariable		Multivariable	
	OR (95% C.I.)	*p*	OR (95% C.I.)	*p*
Moro dif.	3.169 (1.314–7.649)	0.010	2.809 (1.018–7.754)	0.046
GSR dif.	1.456 (0.582–3.642)	0.422	-	-
Age	1.215 (0.939–1.571)	0.138	1.124 (0.843–1.500)	0.425
Gender (M)	0.519 (0.100–2.692)	0.435	0.411 (0.067–2.514)	0.336
Initial Cobb	1.077 (1.008–1.151)	0.029	1.033 (0.952–1.120)	0.441

**Table 10:** ROC curve in establishing the cut-off value for the Moro score evolution difference in scoliosis grading improvement.

Parameter	AUC (95% C.I.)	Std. Error	*p*
**Moro dif.**	0.679 (0.455–0.903)	0.114	0.092

**Table 11:** Correlation between the initial value of the Cobb angle and Moro, ATNR, STNR, and GSR initial values.

Correlation	*p* *
Cobb angle × Moro score	0.057, R = 0.175
Cobb angle × ATNR	0.698, R = −0.036
Cobb angle × STNR	0.582, R = −0.051
Cobb angle × GSR	0.011, R = 0.233

* Spearman’s rho correlation coefficient.

**Table 12:** Correlations between the initial value of the Cobb angle and Moro, ATNR, STNR, and GSR evolution differences.

Correlation	*p* *
Cobb angle × Moro score—Difference	0.028, R = 0.203
Cobb angle × ATNR—Difference	0.690, R = −0.037
Cobb angle × STNR—Difference	0.837, R = −0.019
Cobb angle × GSR—Difference	0.011, R = 0.234

* Spearman’s rho correlation coefficient.

**Table 13:** Comparison of initial values of Moro, ATNR, STNR, and GSR scores according to the existence of quadrupedal locomotion.

Moro score/Quadrupedal locomotion	Mean ± SD	Median (IQR)	Mean Rank	*p* *
Absent	0.31 ± 0.74	0 (0–0)	75.96	0.851
Present	0.25 ± 0.59	0 (0–0)	75.05	
**ATNR score/Quadrupedal locomotion**				
Absent	0.68 ± 0.77	0.5 (0–1)	84.89	0.002
Present	0.33 ± 0.59	0 (0–1)	66.36	
**STNR score/Quadrupedal locomotion**				
Absent	1.09 ± 0.64	1 (1–1)	106.31	<0.001
Present	0.05 ± 0.22	0 (0–0)	45.50	
**GSR score/Quadrupedal locomotion**				
Absent	0.5 ± 0.76	0 (0–1)	82.12	0.017
Present	0.24 ± 0.53	0 (0–0)	69.05	

* Mann-Whitney U test.

## Data Availability

The collected data are part of an ongoing doctoral study and will be available for publication upon the completion of the thesis; they are currently in the archives of the clinic where the study was conducted and are available upon request.

## Abbreviations

The following abbreviations are used in this manuscript:

AIS	Adolescent Idiopathic Scoliosis
PSSE	Physiotherapeutic Scoliosis Specific Exercises
ATNR	Asymmetric Tonic Neck Reflex
STNR	Symmetric Tonic Neck Reflex
SGR	Spinal Galant Reflex
FTI	Finger-to-Toe Index
ISST	International Schroth 3-dimensional Scoliosis Therapy
SOSORT	International Society on Scoliosis Orthopedic and Rehabilitation Treatment
SRS	Scoliosis Research Society
